# Biochar Integrate with Beneficial Microorganisms Boosts Soil Organic Fractions by Raising Carbon-Related Enzymes and Microbial Activities in Coastal Saline-Alkali Land

**DOI:** 10.3390/microorganisms14010115

**Published:** 2026-01-05

**Authors:** Rui Wang, Qian Cui, Zeyuan Wang, Hongjun Yang, Yuting Bai, Ling Meng

**Affiliations:** 1 Shandong Key Laboratory of Eco-Environmental Science for Yellow River Delta, Shandong University of Aeronautics, Binzhou 256603, China; wr1807943517@163.com (R.W.);; 2 Yantai Institute of Coastal Zone Research, Chinese Academy of Sciences, Yantai 264003, China

**Keywords:** biochar, BM, soil organic carbon fraction, microbial community, coastal saline-alkali land

## Abstract

Biochar and beneficial microorganisms (BM) is considered promising soil amendment for saline-alkali amelioration and soil carbon storage.However, the effects of biochar combined with BM addition soil organic carbon (SOC) accumulation and microbial characteristics are less known in coastal saline-alkali soil. Herein, we investigated the SOC content and fractions, soil carbon enzyme activities, and microbial community composition in coastal saline-alkali soil, following three levels of biochar and BM addition. Compared to the control treatment, biochar and BM application effectively reduced soil salinity by 37.58–66.53% and increased soil NH_4_^+^ by 9.49–121.16% and NO_3_^−^ by 43.56–254.28%, respectively. Biochar integrated with BM addition significantly increased the content of SOC, soil mineral-associated organic carbon (MAOC), soil particulate organic carbon (POC), and carbon pool management index (CPMI) by 37.76–108.02%, 15.43–140.44%, 13.73–64.55%, and 81.11–154.61%, respectively, compared with CK treatment. Additionally, biochar and BM significantly enhanced the activities of soil carbon cycle enzymes, including α-1,4-glucosidase (14.54–124.45%), β-1,4-glucosidase (12.71–133.98%), and cellulose hydrolase (6.07–19.17%). Biochar and BM addition also improved the bacterial diversity and altered the microbial composition at the phylum level. The co-addition of biochar and BM improved SOC by decreasing soil salinity and, enhancing soil nutrient availability, soil carbon cycle enzymes, and microbial activity. Furthermore, the combination of 4% biochar and BM exhibited the highest MAOC/POC ratio, demonstrating the most significant impacts on enhancing SOC stability in coastal saline-alkali soil. This study highlighted that the combined use of biochar and BM could serve as a promising approach to fortify soil carbon pool content and stability in saline-alkali land.

## 1. Introduction

Saline-alkali land constitutes approximately 7% of the world’s land reserves and soil salinization has emerged as a major environmental constraint affecting global agricultural productivity [1]. China possesses approximately 36.7 million hectares of utilizable saline-alkali land resources, which hold enormous development potential [2]. Coastal saline-alkali land is widely distributed across China’s coastal regions, primarily resulting from seawater intrusion and high groundwater mineralization [3]. Coastal saline-alkali land is characterized by sparse vegetation cover, poor soil quality, low soil organic carbon (SOC), and restricted land utilization efficiency [4]. SOC serves as a key parameter for assessing soil health, as it mitigates saline-alkali soil degradation by enhancing soil properties, fertility, and microbial activity [5]. Salt-affected soil also exhibits great potential capacity for carbon capture and sequestration [1]. Improving the soil carbon storage of saline-alkali soil is critical for ameliorating soil quality and mitigating global climate change [6]. Therefore, it is imperative to explore effective measures to enhance carbon storage in saline-alkaline lands.

Biochar, a material featuring a porous nature and abundant carbon, has been shown to improve saline-alkali soil environments by modifying the soil’s physical, chemical, and biological characteristics [4,7]. Its significant specific surface area and the existence of diverse functional groups help enhance water retention and cation exchange capacity, improve soil structure and microbial activity, and decrease bulk density, thus alleviating salt stress [8]. Biochar application is an efficient measure for enhancing SOC sequestration in the soil–biochar mixture [9], which offers numerous environmental and economic advantages to ecosystems [6]. The mechanisms responsible for the accumulation of SOC have predominantly been ascribed to the high stable SOC and the labile SOC fractions from the improved soil microbiota and nutrient cycling [10]. Recent meta-analyses have shown that biochar can improve SOC reserves at a rate of 0.91 Mg C ha^−1^ year^−1^ in Chinese farmland, with pronounced impacts on long-term carbon sequestration [11]. Biochar addition can increase greater carbon input into the soil by boosting plant development and productivity [12]. BM inoculants have attracted a lot of attention as a co-friendly modification strategy in enhancing soil fertility and microbial activities, improving plant growth, and mitigating soil salinity stress [13,14]. Contrasted with the single-strain microbial inoculants, BM encompasses a wide variety of beneficial microorganisms [15,16], leading it to multiply microbial proliferation and promote dominant microbial populations [13]. Biochar can serve as an effective carrier for BM, enhancing their colonization and metabolic activities in the soil environment. The integration of biochar with BM can generate synergistic effects, significantly enhancing soil microbial activity and soil nutrient availability, thereby promoting soil carbon storage and plant productivity [4,14]. However, the underlying mechanism of how biochar and EM affect SOC stability by regulating soil nutrient and microbial properties, as well as soil enzyme activities, remains to be ascertained and warrants further investigation.

Biochar has been widely recognized for its significant impact on the contents, ratios, and dynamic equilibrium of SOC components [10,17]. The SOC pool is divided into particulate organic carbon (POC) and mineral-bound organic carbon (MAOC), both of which are crucial for the cycling and transformation of soil nutrients [18]. Biochar facilitates the formation of soil aggregates and pore structures [19], providing physical protection that promotes POC and MAOC fixation [10]. Microbial biomass carbon (MBC) and dissolved organic carbon (DOC), integral components of the soil organic carbon (SOC) pool, serve as critical and highly reactive fractions within the labile SOC. The findings of the meta-analysis showed that biochar significantly increased SOC, MBC, and labile carbon by 84.3%, 20.1%, and 22.9%, respectively, driven by the stimulated microbial growth and activity [12]. Biochar provides a large surface area and abundant pore structures, that serve as favorable habitats for soil microorganisms. Biochar promotes DOC mineralization by altering soil microbial communities [20], resulting in the improvement in MBC and soil fertility [6,18]. Meanwhile, soil enzymes are the catalysts of soil organic matter (SOM) decomposition and are involved in the microbial activities. Biochar application to soil has optimistic effects on soil carbon cycle enzyme activities [21,22], thus promoting microbial activities and nutrient supply, altering SOC fractions, and enhancing carbon sequestration [23]. However, existing research has demonstrated that biochar amendments have no effect or a negative effect on SOC and SOC components [12]. These conflicting results can be attributed, in part, to the intricate interaction among the heterogeneous characteristics of biochar, different soil types, various management practices, and diverse climatic conditions [10,12].

The Yellow River Delta, a well-known coastal saline-alkali region in China, is particularly affected by severe soil salinization, with over 85% of its land exhibiting varying degrees of salinity [24]. Applying biochar and BM has been a green strategy to reduce salinity stress, improve soil quality, and ensure crop production in this region [4,14]. However, the impact and mechanisms of biochar and BM on SOC sequestration remain unclear. In this study, a pot experiment was conducted to assess the combined impacts of biochar and BM on various soil carbon fractions, carbon cycle enzyme activities, and bacterial diversity. The objectives were as follows: (1) to quantify the changes in soil properties, carbon cycle enzyme activities, and bacterial community following biochar and BM addition, (2) to compare the influence of biochar addition individually or combined with BM on different SOC fractions, their individual contribution percentages, and CPMI, (3) to elucidate the biological mechanisms of SOC sequestration after adding biochar and BM. We hypothesized that biochar integrated with BM addition increased the sequestration of SOC through soil nutrient, carbon cycle enzyme activities, bacterial community, and SOC fractions.

## 2. Materials and Methods

### 2.1. Soil and Biochar Preparation

The coastal saline-alkali soil samples were obtained from Kenli District, Dong Ying City, Shandong Province (37°34′ N and 118°31′ E), in April 2024. Surface soil (0–20 cm) was randomly collected at five points and the five soil samples were mixed into a homogeneous composite. Soil samples were then transported to the laboratory and air-dried, mechanically pulverized, and sieved with a 2 mm mesh to obtain uniformly sized particles. The soil physical and chemical properties were analyzed, showing pH at 7.53, a salt content at 0.62%, SOC at 9.26 g·kg^−1^, total nitrogen (TN) at 0.44 g·kg^−1^, available phosphate (AP) at 3.8 mg·kg^−1^.

Corn straws were selected as raw material for biochar production because of low cost, environmentally friendly nature, and efficient utilization of biomass energy, which is widely used in saline-alkali soil. Biochar was produced by corn stalks under oxygen-limited conditions at 500 °C for 2 h. The biochar sample was pulverized and passed through a sieve with 100-mesh, then kept in a Ziploc bag for later use. Biochar exhibited a total carbon content of 67.49%, a total nitrogen content of 1.65%, a hydrogen content of 4.84%, and an oxygen content of 19.51%. It possessed a carbon-to-hydrogen ratio of 40.90, a particular surface area of 34.85 m^2^·g^−1^, and a pH level of 7.95. Based on the standardized criteria and norms, BM inoculants primarily consist of 3.3 × 10^4^ cfu·mL^−1^ photosynthetic bacteria, 1.3 × 10^7^ cfu·mL^−1^ lactic acid bacteria, 10^5^ cfu·mL^−1^ fermenting fungi, and 1.3 × 10^4^ cfu·mL^−1^ yeast and various other bacteria.

### 2.2. Experiment Design

The pot experiment was designed with eight treatments organized in a factorial design with two factors, featuring four replicates for each treatment. The treatments included CK (control), CSB1 (1% biochar), CSB2 (2% biochar), CSB3 (4% biochar), BM (BM addition), CSB1 + BM (1% biochar with BM), CSB2 + BM (2% biochar with BM), CSB3 + BM (4% biochar with BM). The BM solution was diluted with deionized water to a concentration of 0.5. Each BM-treated pot received 4 mL·pot^−1^ solution and the untreated pot received double distilled water at the time of irrigation. Polyethylene containers with an external diameter of 15 cm, internal diameter of 13 cm, and height of 12 cm were used in the experiment. Each container held 500 g soil, and soil moisture level was maintained at 60% to 70% of the field’s water holding capacity. Alfalfa seeds with full grains and no spots were selected, sterilized with 10% H_2_O_2_ for 0.5 h, soaked in saturated CaSO_4_ for 12 h, and then rinsed with distilled water before sowing, with 15 seeds in each pot. Two weeks after sowing, the seedlings were inter-seeded and six alfalfa plants were retained in each pot. The pot experiment was conducted under controlled greenhouse conditions, with the bottom of each container equipped with a tray to collect water that was lost by irrigation. Each plot was watered regularly, and weeds were cleared away to guarantee normal growth. In May 2024, the alfalfa was sown and the harvest was completed by September of the same year. After harvesting, soil samples from each container were taken. Soil was sieved and divided into three sections, one was stored at −4 °C for soil fertility and SOC fractions analysis, one was kept at −80 °C for assessing soil enzyme activities, and another was immediately stored at −80 °C to measure microbial activity.

### 2.3. Soil Properties and Soil Enzyme Activities

Soil pH was measured using a pH meter (FE22-Standard, METTLER TOLEDO, Shanghai, China) with a soil-to-water ratio of 1:5. Soil salinity was determined by the residue weight method after drying. This involved preparing a soil–water extract (soil-to-water ratio: 1:5), evaporating the supernatant to concentrate it, drying the residue at 105–110 °C to constant weight, and calculating the total salt percentage based on the residue mass. Total carbon (TC) and TN in the soil were quantified using an elemental analyzer(Elementar, Hanau, Germany). The Olsen method was employed to analyze AP, and available potassium (AK) was assessed using NH_4_Ac extraction followed by flame photometry. Concentrations of NH_4_^+^ and NO_3_^−^ in the soil were identified with an AA3 automated flow injection analyzer from SEAL Analytics GmbH in Norderstedt, Germany. The activities of α-1,4-glucosidase (AG), β-1,4-glucosidase (BG), β-Xylosidase (XG), and cellulose hydrolase (CBH) in soil were determined using a fluorescent substrate microplate assay. AG, BG, XG, and CBH were measured using 4-MUF-α-D-glucopyranoside, 4-MUF-β-D-glucopyranoside, 4-MUF-β-D-xylopyranoside, and 4-MUF-β-D-cellobiose as substrates, respectively. The final concentration of all substrates was set at 500 µmol·L^−1^. The reaction system was incubated at an appropriate temperature (e.g., 30 °C or 37 °C) for 1 h (60 min), followed by addition of a stop solution to terminate the reaction. The fluorescence intensity of the product 4-MU was measured using a fluorometer(Meixi Instruments, Shanghai, China), and the final enzyme activity was expressed as nmol·kg^−1^·h^−1^ [25,26].

### 2.4. Soil Organic Carbon Content and Fractions

The dichromate oxidation technique was utilized to assess SOC, with POC and MAOC segments determined following Sokol et al. [27]. In brief, soil organic carbon fractionation was conducted by dispersing 20 g of air-dried soil in 60 mL sodium hexametaphosphate (5 g·L^−1^) via 18 h horizontal shaking, followed by size selective wet-sieving through a 53-μm mesh to isolate POC fraction (>53 μm) and MAOC fraction (<53 μm). These portions were dried at 65 °C and screened using a 0.15 mm sieve for SOC analysis. DOC and MBC were measured based on the method developed by Dai et al. [28]. Active carbon (AC) was oxidized using 333 mmol·L^−1^ KMnO_4_, while inert carbon (RC) was computed by subtracting AC from SOC.

The CPMI in different treatments was calculated using CK treated soil as the reference. The carbon pool index (CPI), lability of C (CL), lability index (LI), and CPMI were calculated according to the method of Blair et al. (1995) [29]. The calculation is as follows:

(1) CPMI=CPI×LI×100

where $CPI=\frac{SOC_{i}(g\cdot kg^{-1})}{SOC_{ck}(g\cdot kg^{-1})}$, $CL=\frac{AC(g\cdot kg^{-1})}{RC(g\cdot kg^{-1})}$, and $L=\frac{{CL}_{i}}{CL_{ck}}$.

The *SOC_i_* and *SOC_CK_* represent the SOC content of different treatments and the control treatment, respectively. *CL_i_* and *CL_CK_* represent the CL value of different treatments and the control treatment, respectively.

### 2.5. Soil Bacterial Diversity

Total genomic DNA was extracted from samples using the CTAB method. DNA quality was confirmed by 1% agarose gel electrophoresis, and the DNA was diluted to 1 ng/µL with sterile water. The V3–V4 hypervariable regions of the 16S rRNA gene were amplified using specific primers 341F (5′-CCTAYGGGRBGCASCAG-3′) and 806R (5′-GGACTACNNGGGTATCTAAT-3′) with barcodes. PCR was performed using Phusion^®^ High-Fidelity PCR Master Mix (New England Biolabs, Ipswich, MA, USA) under the following conditions: initial denaturation at 98 °C for 1 min, followed by 30 cycles of 98 °C for 10 s, 50 °C for 30 s, and 72 °C for 30 s, and a final extension at 72 °C for 5 min. The resulting PCR products were purified using a Universal DNA purification kit (Tiangen, Tianjin, China) after detection on a 2% agarose gel. Sequencing libraries were prepared using the NEB Next® Ultra DNA Library Prep Kit (Illumina, San Diego, CA, USA) and sequenced on an Illumina platform to generate 250 bp paired-end reads.

Bioinformatics analysis followed the “Atacama soil microbiome tutorial” from Qiime2docs (2023.9) [30]. Raw FASTQ data were imported into QIIME2 (2023.9), and the DADA2 (1.26.0) plugin was used for quality filtering, trimming, de-noising, merging, and chimera removal to obtain amplicon sequence variants (ASVs) [31]. Taxonomic assignment was performed using the QIIME2 feature-classifier plugin against a pre-trained GREENGENES 13_8 99% database, trimmed to the V3–V4 region. Contaminating mitochondrial and chloroplast sequences were subsequently filtered out. Microbial diversity was assessed using the QIIME2 core-diversity plugin. Alpha diversity indices, including Chao1 richness and Shannon diversity, were calculated to estimate within-sample diversity.

### 2.6. Statistical Analysis

The gathered experimental results were meticulously arranged and assessed through Microsoft Office Excel 2024. To evaluate the impact of biochar, BM, and their interplay on soil properties and nutrients, SOC pool fractions, proportions of SOC pool fractions, and soil carbon cycle enzymes, a two-way ANOVA accompanied by Tukey’s test was employed. To examine the differences in soil properties and nutrients, SOC pool fractions, proportions of SOC pool fractions, soil carbon cycle enzymes, and microbial community diversity and composition between different treatment samples, a one-way ANOVA was conducted utilizing the SPSS 27.0 program (IBM Corp., Armonk, NY, USA), followed by Tukey’s test (*p* < 0.05). The composition of the microbial community was depicted through non-metric multidimensional scaling (NMDS) utilizing Bray–Curtis distance metrics. To explore possible correlations among SOC, POC, MAOC, MBC, DOC, RC, soil properties, enzyme activities, bacterial community structure, and diversity indices, Pearson correlation analysis was conducted using Origin software (version 2024b). The partial least squares structural equation model (PLS-SEM) was constructed utilizing the ‘PLSPM’ package within R software (version 4.5.0) and accessed via the R Studio platform (2022.07.2+576) to elucidate the impact of biochar and BM addition on POC, MAOC, and SOC. Graphical combinations were created using Adobe Photoshop software (version 25.9.0).

## 3. Results

### 3.1. Variations in Soil Properties and Soil Nutrients

**Figure 1:**
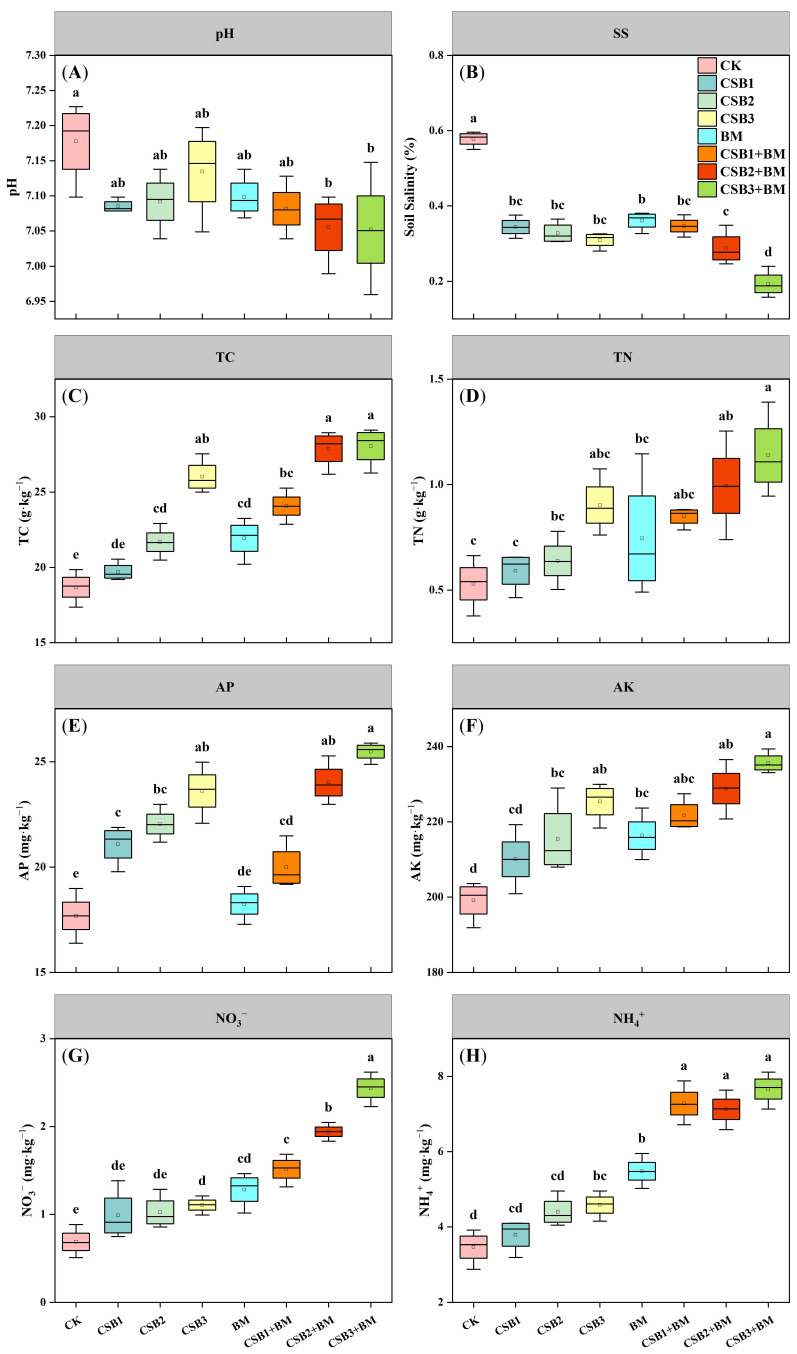
Effects of different treatments on pH (**A**), soil salinity (**B**), TC (**C**), TN (**D**), AP (**E**), AK (**F**), NO_3_^−^ (**G**), and NH_4_^+^ (**H**) of soil (mean ± SE, *n* = 4). CK, control; CSB1, 1% biochar; CSB2, 2% biochar; CSB3, 4% biochar; BM, BM addition; CSB1 + BM, 1% biochar with BM; CSB2 + BM, 2% biochar with BM; CSB3 + BM, 4% biochar with BM. Different lowercase letters show significant differences under different treatments (*p* < 0.05, Tukey’s test).

Biochar and BM application significantly influenced soil salinity, TC, TN, AP, AK, NH_4_^+^, and NO_3_^−^ concentrations (*p* < 0.05). However, the interactive effects of biochar and BM were statistically significant only for soil salinity, TC, AP, NH_4_^+^, and NO_3_^−^ concentrations (Table 1A, *p* < 0.05). Notably, significant differences in soil pH were only observed under CSB2 + BM and CSB3 + BM treatment in comparison with the CK treatment (Figure 1A, *p* < 0.05). Specifically, the combined utilization of biochar and BM resulted in a 37.58–66.53% reduction in salinity relative to the CK treatment, showing the most significant decrease under the CSB3 + BM treatment (Figure 1B). In contrast to the CK treatment, the CSB3 + BM treatment also led to a 50.13% increase in TC (Figure 1C). While biochar amendments alone increased TN by 11.61–70.37%, the addition of BM further enhanced this effect, resulting in increases ranging from 60.12 to 114.69% (Figure 1D). Significant increases in AP (3.20–44.07%) and AK (5.49–8.35%) were also observed following biochar and BM application, as well as their interaction (Figure 1E,F). In comparison with the CK treatment, the CSB3, BM, CSB1 + BM, CSB2 + BM, and CSB3 + BM treatments significantly elevated soil NO_3_^−^ and NH_4_^+^ by 60.77% to 254.28% and 32.22% to 121.16%, respectively (Figure 1G,H, *p* < 0.05). The enhancement in soil NO_3_^−^ response to biochar and BM was much stronger than that of soil NH_4_^+^.

### 3.2. Variations in SOC, SOC Pool Fractions, and CPMI

**Figure 2:**
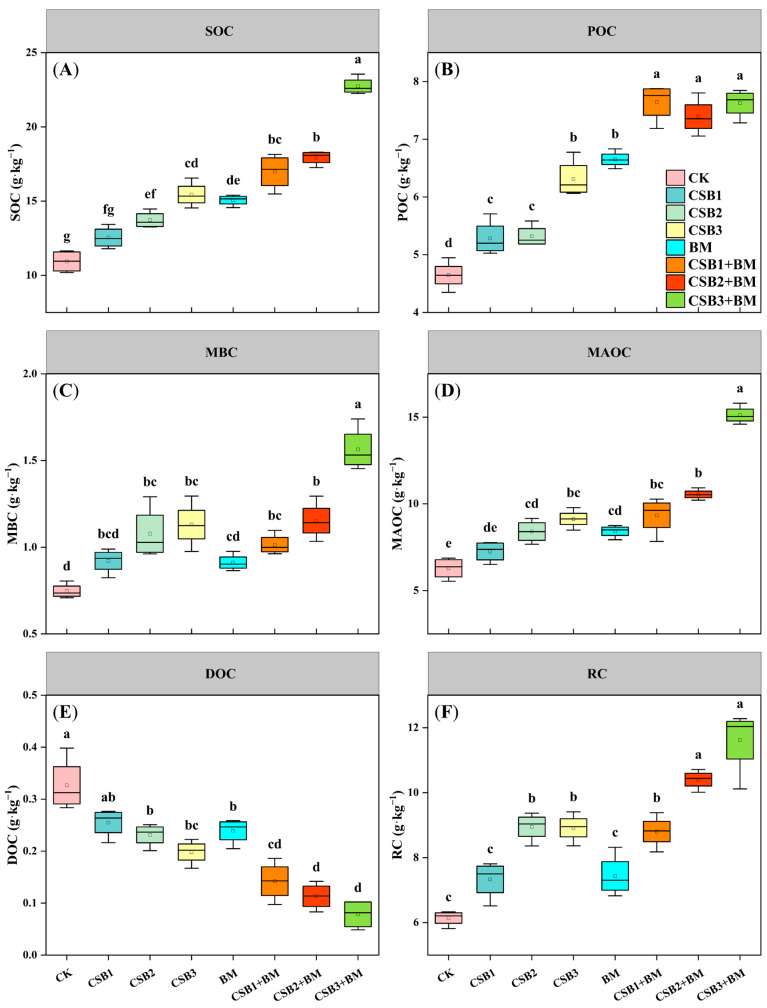
Changes in SOC (**A**), POC (**B**), MBC (**C**), MAOC (**D**), DOC (**E**), and RC (**F**) contents among different treatments (mean ± SE, *n* = 4). CK, control; CSB1, 1% biochar; CSB2, 2% biochar; CSB3, 4% biochar; BM, BM addition; CSB1 + BM, 1% biochar with BM; CSB2 + BM, 2% biochar with BM; CSB3 + BM, 4% biochar with BM. Different lowercase letters show significant differences under different treatments (*p* < 0.05, Tukey’s test).

Biochar and BM, when applied separately, had notable impacts on soil SOC, POC, MBC, MAOC, DOC, and RC (*p* < 0.001). However, the combined effect of biochar and BM significantly influenced only soil SOC, POC, MBC, and MAOC (Table 1B, *p* < 0.01). Furthermore, the changes observed in SOC, POC, MBC, MAOC, DOC, and RC were not entirely consistent across treatments. The contents of SOC, POC, MBC, MAOC, and RC all increased significantly with increasing biochar application rates (Figure 2, *p* < 0.05). Specifically, relative to the CK treatment, SOC content increased from 14.71% (CSB1) to 108.02% (CSB3 + BM) (Figure 2A). SOC is divided into POC and MAOC components; MAOC content consistently exceeded POC content across all treatment groups. With increasing biochar application, both POC and MAOC contents exhibited significant increases (Figure 2B,D, *p* < 0.05). Compared to the CK treatment, POC content increased by 13.73%, 14.48%, 35.91%, 43.18%, 64.55%, 59.13%, and 64.13% in the CSB1, CSB2, CSB3, BM, CSB1 + BM, CSB2 + BM, and CSB3 + BM, respectively. Similarly, MAOC content increased by 15.43%, 33.58%, 45.10%, 33.76%, 48.43%, 67.58%, and 140.44% in the same respective treatments. Significant increases in both MBC and RC were exclusively observed in the CSB2, CSB3, CSB1 + BM, CSB2 + BM, and CSB3 + BM treatments, compared to the CK treatment (*p* < 0.05). The most pronounced enhancement was seen in CSB3 + BM treatment, which boosted MBC by 109.48% and RC by 89.21% (Figure 2C,F). In contrast to the other carbon fractions, DOC content decreased significantly with increasing biochar addition, ranging from a decrease of 21.94% (CSB1) to 76.01% (CSB3 + BM) compared to the CK treatment (Figure 2E).

**Figure 3:**
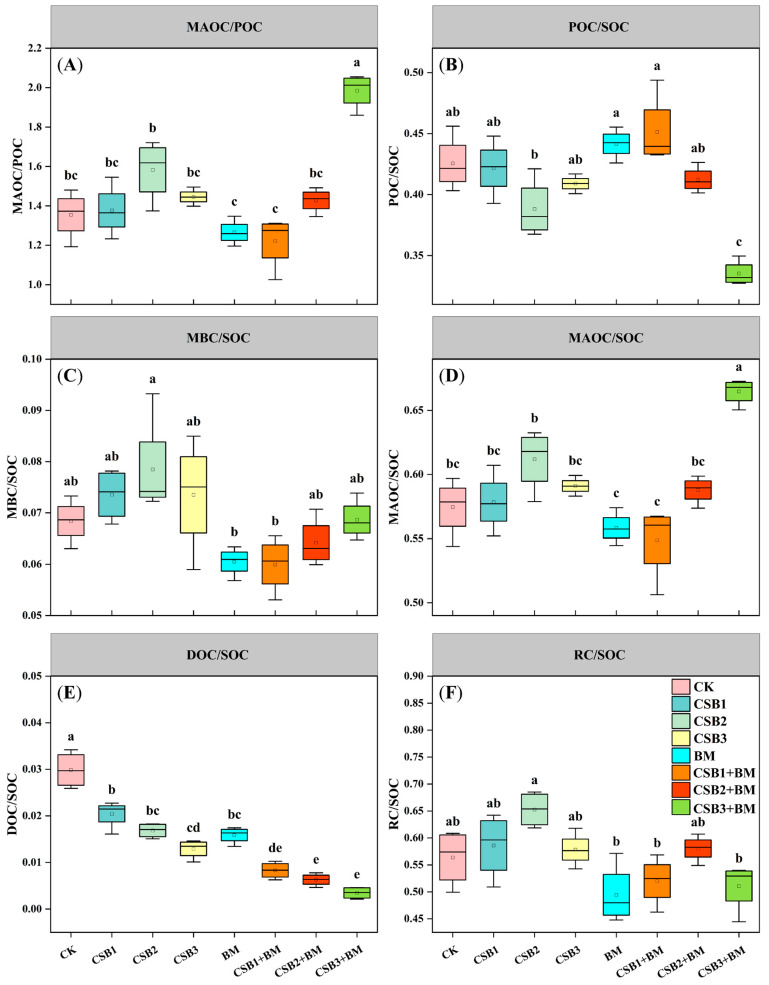
Changes in MAOC/POC (**A**), POC/SOC (**B**), MBC/SOC (**C**), MAOC/SOC (**D**), DOC/SOC (**E**), and RC/SOC (**F**) among different treatments (mean ± SE, *n* = 4). CK, control; CSB1, 1% biochar; CSB2, 2% biochar; CSB3, 4% biochar; BM, BM addition; CSB1 + BM, 1% biochar with BM; CSB2 + BM, 2% biochar with BM; CSB3 + BM, 4% biochar with BM. Different lowercase letters show significant differences under different treatments (*p* < 0.05, Tukey’s test).

Biochar individually exerted significant influences on soil MAOC/POC, POC/SOC, MAOC/SOC, DOC/SOC, and RC/SOC. BM individually exerted significant influences on soil MBC/SOC, DOC/SOC, and RC/SOC. However, the effect of biochar combined with BM was only significant for soil MAOC/POC, POC/SOC, and MAOC/SOC ratios (Table 1C). The MAOC/POC ratio, a key indicator of soil carbon pool stability and carbon cycle dynamics, exhibited a significant fluctuating trend, initially increasing, then decreasing, and subsequently increasing again across different treatments (Figure 3A, *p* < 0.05). The soil POC/SOC ratio decreased with increasing biochar application rates (Figure 3B). Similarly, without BM addition, soil MBC/SOC and MAOC/SOC increased with increasing biochar application. This positive correlation was also observed with BM application (+BM) (Figure 3C,D). In contrast to the CK treatment, biochar alone, BM alone, and their co-application led to a significant reduction in the soil DOC/SOC ratio, with the reduction ranging from 31.56% to 88.40% (Figure 3E). The CSB2 treatment exhibited the highest soil RC/SOC ratio compared to the CK treatment, whereas the BM treatment alone resulted in the lowest RC/SOC ratio (Figure 3F).

In contrast to the CK treatment, CSB3 + BM increased CL and LI by 24.36% and 24.00%, respectively. BM alone increased CL and LI by approximately 33.33% and 32.00%, respectively. The highest increase occurred in the CSB3 + BM treatment, where CPI reached 2.08 ± 0.03, representing a doubling of 108.00% relative to the CK treatment. Other CSB + BM combination treatments (CSB1 + BM and CSB2 + BM) also achieved substantial increases of 55.00% and 64.00%, respectively. The CSB3 + BM treatment yielded the optimal value, with a CPMI of 257.23 ± 25.5, representing a substantial 154.61% increase compared with the CK treatment (101.03 ± 14.13). Other CSB + BM combination treatments, such as CSB1 + BM and CSB2 + BM, also achieved increases of 84.06% and 50.21%, respectively (Table 2).

### 3.3. Variations in Soil Carbon Cycle Enzymes

**Figure 4:**
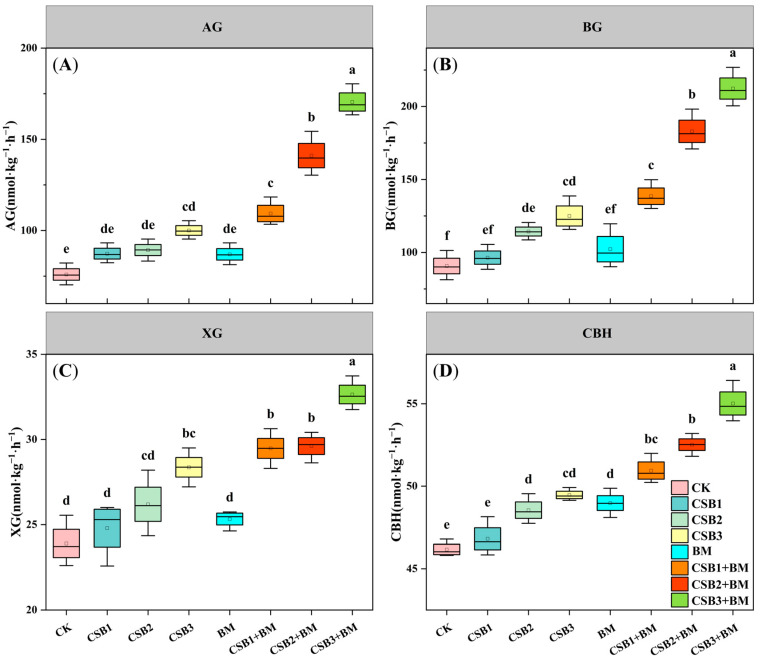
Changes in AG (**A**), BG (**B**), XG (**C**), and CBH (**D**) among different treatments (mean ± SE, *n* = 4). CK, control; CSB1, 1% biochar; CSB2, 2% biochar; CSB3, 4% biochar; BM, BM addition; CSB1 + BM, 1% biochar with BM; CSB2 + BM, 2% biochar with BM; CSB3 + BM, 4% biochar with BM. Different lowercase letters show significant differences under different treatments (*p* < 0.05, Tukey’s test).

Two-way ANOVA revealed that both biochar and BM and their interactive effect exerted significant effects on AG, BG, XG, and CBH activities (Table 1D, *p* < 0.05). Relative to the CK treatment, AG content increased in the CSB1 (15.03%), CSB2 (17.67%), CSB3 (31.76%), BM (14.54%), CSB1 + BM (44.05%), CSB2 + BM (85.77%), and CSB3 + BM (124.45%) treatments (Figure 4A). Similarly, BG content also increased in these respective treatments by 6.32%, 26.03%, 37.77%, 12.71%, 52.69%, 101.67%, and 133.98% (Figure 4B). Compared with the CK treatment, only the CSB3, CSB1+BM, CSB2 + BM, and CSB3 + BM treatments led to significant differences in XG content (*p* < 0.05), increasing by 18.68% to 36.56% (Figure 4C). In comparison with the CK treatment, the CBH content significantly increased in the CSB2 (5.14%), CSB3 (7.14%), BM (6.07%), CSB1 + BM (10.35%), CSB2 + BM (13.75%), and CSB3 + BM (19.17%) treatments (Figure 4D, *p* < 0.05).

### 3.4. Variations in Soil Bacterial Community Diversity

**Figure 5:**
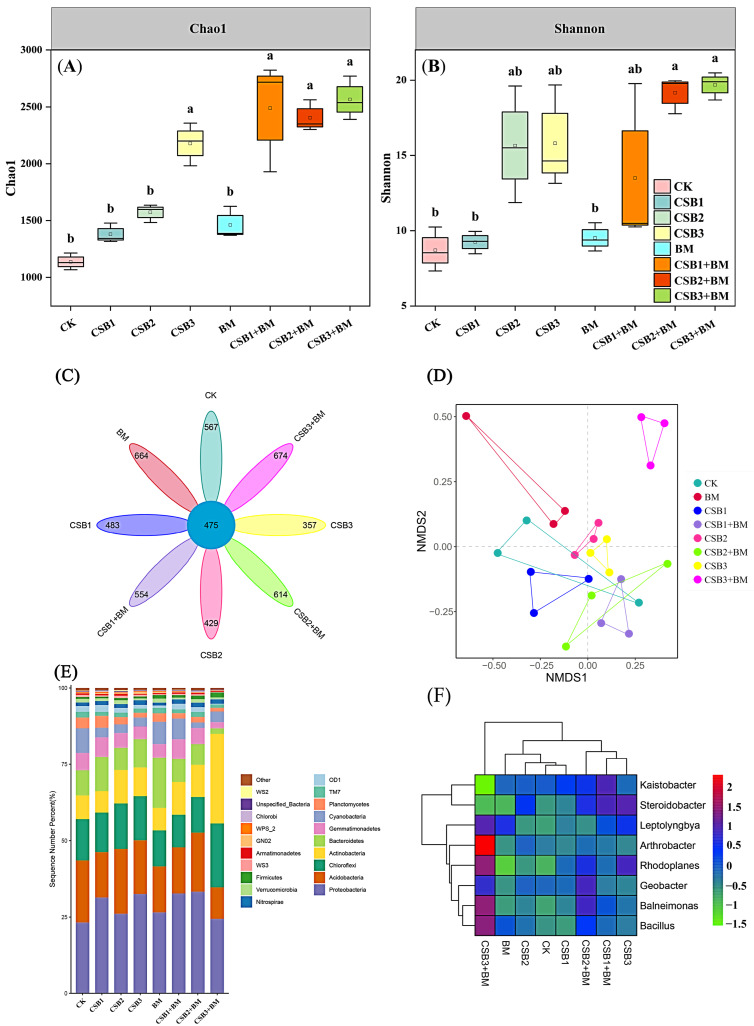
Microbial analysis at different treatments: Chao1 index (**A**), Shannon index (**B**), Venn diagram (**C**), and NMDS (**D**). CK, control; CSB1, 1% biochar; CSB2, 2% biochar; CSB3, 4% biochar; BM, BM addition; CSB1 + BM, 1% biochar with BM; CSB2 + BM, 2% biochar with BM; CSB3 + BM, 4% biochar with BM. Bacterial community structures: top 20 bacteria at phylum level (**E**) and top 8 bacteria at genus level (**F**). Different lowercase letters show significant differences under different treatments (*p* < 0.05, Tukey’s test).

The application of biochar alone, BM alone, and their combination led to a statistically significant rise in both the Chao1 index and the Shannon index of the soil bacterial community (*p* < 0.05), demonstrating the positive influence of these treatments on microbial community structure (Figure 5A,B). Notably, the CSB3 + BM treatment exhibited the highest Chao1 and Shannon indices, suggesting that biochar combined with BM addition is especially efficient in enhancing bacterial community diversity and richness. This effect may be attributed to biochar providing a suitable habitat and BM supplying readily available organic matter and inherent microbial diversity. A total of 8142 operational taxonomic units (OTUs) were identified across all treatments, with only 475 OTUs shared among all groups. The CSB3 + BM treatment exhibited the highest number of unique OTUs (1149) (Figure 5C). Non-metric multidimensional scaling (NMDS) analysis demonstrated distinct variations in the microbial communities across the different treatments, with a stress value of < 0.1 confirming the reliability of the ordination (Figure 5D). The proximity of the CK treatment to the single biochar treatment group, in contrast to the CSB3 + BM treatment, which was more distant from the CK treatment, suggests that BM contributed more substantially to alterations in the soil microbial community structure.

*Proteobacteria* dominated the phylum-level hierarchy, with *Acidobacteria*, *Chloroflexi*, *Actinobacteria*, and *Bacteroidetes* sequentially comprising the core microbiome (Figure 5E). All treatments significantly elevated *Proteobacteria* abundance relative to the CK treatment (*p* < 0.05), with the highest increase observed in the CSB2 + BM treatment. Notably, *Actinobacteria* and *Chloroflexi* were most abundant under the CSB3 + BM treatment, representing increases of 282.66% and 53.23%, respectively. Conversely, the relative abundance of *Acidobacteria* and *Bacteroidetes* was lowest in the CSB3 + BM treatment, decreasing by 49.25% and 78.58%, respectively, compared to the CK treatment. Genera *Bacillus*, *Balneimonas*, *Rhodoplanes*, and *Arthrobacter* were significantly enriched in the CSB3 + BM treatment , demonstrating high abundance and increasing by 486.05%, 147.96%, 247.91%, and 292.12%, respectively, relative to the CK treatment (Figure 5F). The CSB2 + BM treatment resulted in the highest abundance of *Geobacter*, which exhibited notable differences across all treatments.

### 3.5. Causality Relationships Between Soil Carbon Fractions, Soil Quality, and Microbial Community

**Figure 6:**
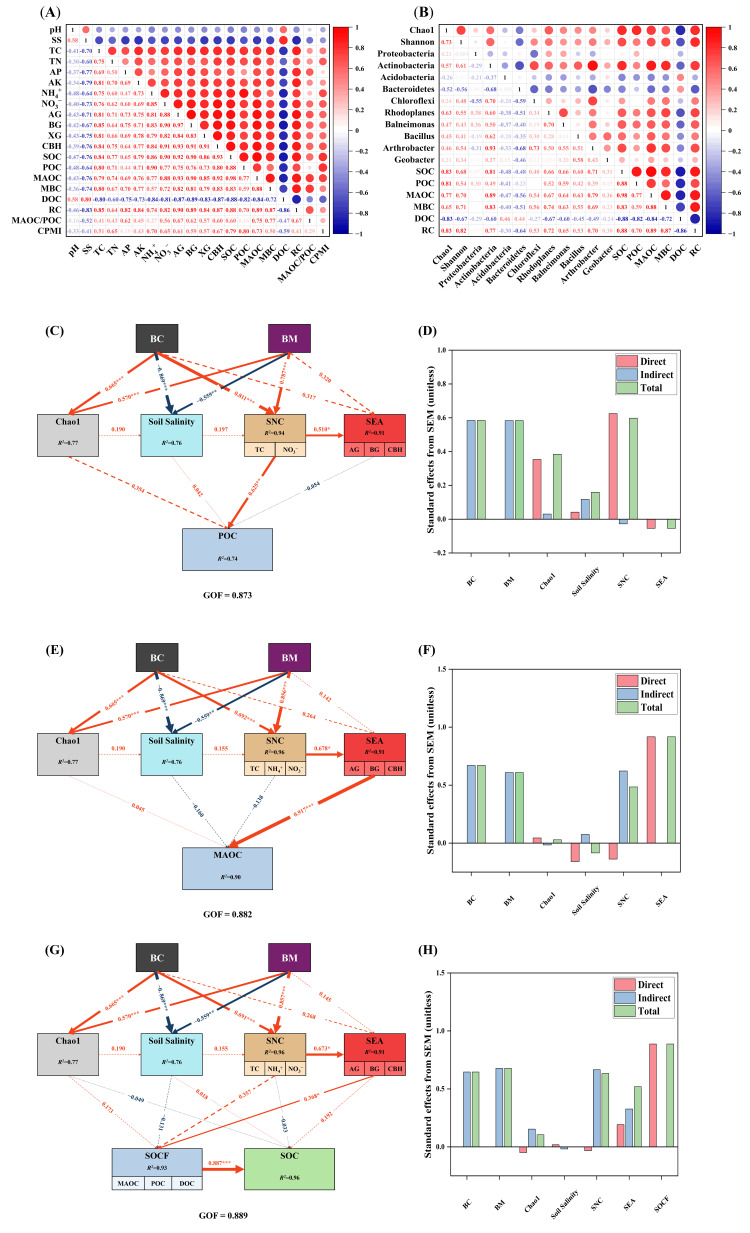
Correlation of soil carbon fractions, soil quality, and microbial community (**A**,**B**). The heat map color represents the Pearson correlation coefficient. The partial least squares structural equation model (PLS-SEM) described the effects of BC, BM, Chao1 index, Soil Salinity, SNC, and SEA on POC (**C**), MAOC (**E**), and SOC (**G**). The solid line indicates a significant relationship, and the dotted line indicates an insignificant relationship. The numbers on the arrows are the path coefficients and are the standardized effect size of the relationships. The thickness of the line represents the size of the path coefficient. Continuous red and blue arrows indicate significant positive and negative relationships (*p* < 0.05), respectively. R^2^ represents the proportion of variance explained. The direct, indirect, and total standardized effects of predictors on POC (**D**), MAOC (**F**), and SOC (**H**) are presented. SNC (soil nutrient content); SEA (soil carbon cycle enzymes); SOCF (soil organic carbon fractions); TC (total carbon); AG (α-1,4-glucosidase); BG (β-1,4-glucosidase); CBH (cellulose hydrolase); MAOC (mineral-associated organic carbon); POC (particulate organic carbon); DOC (dissolved organic carbon); SOC (soil organic carbon). Asterisks indicate significance levels: *** *p* < 0.001, ** *p* < 0.01, * *p* < 0.05.

The Pearson correlation analysis was employed to elucidate the relationships between soil properties and nutrients, SOC pool fractions, MAOC/POC, CPMI, soil carbon cycle enzymes, and bacterial community structure. A significant positive linkage was found between SOC and TC, NH_4_^+^, and NO_3_^−^, AG, BG and CBH, POC, MAOC, and RC, while a negative linkage was found between SOC, soil salinity, and DOC (Figure 6A). Furthermore, SOC exhibited a significant positive correlation with the Chao1 index, *Actinobacteria*, and *Arthrobacter* (Figure 6B).

PLS-SEM was employed to examine the hypothesized relationships and potential underlying mechanisms between BC and BM and POC, MAOC, and SOC (Figure 6C–H). The model fit results indicated that all three models exhibited good agreement with the observed data (GOF = 0.876, 0.882, and 0.889, respectively). Specifically, the models explained 74% of the variance in POC, 90% of the variance in MAOC, and 96% of the variance in SOC. Path analysis results indicated that BC and BM exerted significant positive direct effects on SNC (TC, NO_3_^−^) (path coefficient = 0.811, *p* < 0.001; path coefficient = 0.787, *p* < 0.001), which in turn indirectly influenced POC (path coefficient = 0.811, *p* < 0.001); BC and BM exerted significant positive direct effects on SNC (TC, NO_3_^−^) (path coefficient = 0.692, *p* < 0.001; path coefficient = 0.856, *p* < 0.001), which in turn exerted significant positive effects on SEA (AG, BG, and CBH) (path coefficient = 0.678, *p* < 0.05), ultimately influencing MAOC (path coefficient = 0.917, *p* < 0.001); BC and BM primarily exerted a significant positive direct effect on SNC (TC, NO_3_^−^) (path coefficient = 0.691, *p* < 0.001; path coefficient = 0.857, *p* < 0.001), which in turn exerted a significant positive effect on SEA (AG, BG, and CBH) (path coefficient = 0.673, *p* < 0.05), and subsequently exerted a significant positive influence on SOCF (MAOC, POC, and DOC) (path coefficient = 0.368, *p* < 0.05), ultimately influencing SOC (path coefficient = 0.887, *p* < 0.001).

## 4. Discussion

### 4.1. Modulation of Soil Salinity and Fertility by Biochar and BM Amendments

The application of biochar and BM individually had minimal impact on soil pH, attributing to the degree of saline-alkali soil buffering ability to counteract pH alterations caused by external factors. Interestingly, the combined high-rate use of biochar and BM decreased pH; such a decline may be attributed to the organic acids in BM which accelerate the release of acidity in biochar for alleviating soil basicity [14]. Additionly, the Ca^2+^ and Mg^2+^ ions in saline-alkali soil exchanged with the carboxyl functional group on the surface of biochar, replacing the H of the carboxyl functional group, resulting in a decrease in pH. Our findings demonstrated that soil salinity content was reduced by 37.58% to 66.53% through either individual biochar application or its combination with BM, with the synergistic interaction of biochar and BM yielding significantly greater desalination effects. This result concurs with the prior research that biochar markedly decreased soluble salt by 11.7–54.6% during three years of a biochar application field experiment [32]. Biochar has a significant absorbing ability, owing to the significance of its specific surface area, well-developed porous architecture, and abundant microbial microorganisms. The favorable microorganisms present in BM demonstrated a promising capacity to mitigate soil salinity [13]. Thus, biochar integrated with BM decreased soil salinity, partly because the improved soil structure and hydraulic conductivity reduced the upward migration and buildup of salt in the surface soil [7].

Multiple lines of existing evidence have shown that biochar addition can ameliorate saline-alkali soil by increasing soil fertility and improving nutrient availability [4,33,34]. In this study, biochar and BM, individually or in combination, significantly increase soil TC, TN, AP, and AK, which corroborates the conclusions of Wang et al. and Liu et al. [14,35]. It can be reasonably inferred that biochar is rich in plentiful nutrients, which can provide nutrient sources, water, and habit for enzyme activities and microbial diversity [36]. Specifically, this was supported by the soil enzyme activities and microbial diversities, which exhibited a positive correlation with soil TC, TN, AP, and AK in biochar amended soil. Another reason was that biochar improved soil porosity and increased soil aggregates stability and water holding capacity, potentially boosting the preservation and uptake of soil nutrients [10]. The large specific surface area and high absorption capacity were the reasons that biochar absorbs and releases nutrients, thus increasing soil fertility. Notably, biochar in combination with BM presented greater effects on soil nutrient concentrations, with the CSB3 + BM treatment showing the best effects among all treatments in our study. These results further elucidated the effect of biochar combined with BM on enhancing soil fertility on coastal saline-alkali soil. BM addition could boost microbial and enzymatic activities, which accelerated SOM turnover and produced more soil nutrients [13]. The beneficial microorganisms in BM were capable of synthesizing a variety of bio-active substances that promoted the formation of soil aggregates and diminished nutrient depletion [16]. We observed that biochar combined with BM produced higher NH_4_^+^ and NO_3_^−^ contents, which might be ascribed to the increased transformation of NH_4_^+^ and NO_3_^−^ by the enhanced enzyme activities and SOC availability [14]. This was confirmed by the positive relationship between NH_4_^+^ and NO_3_^−^ and SOC and soil carbon cycle enzyme activities. Moreover, the integrated use of biochar and BM was found to promote Alfalfa biomass by 71.91–126.92%, possibly due to reducing the soil salinity and increasing the nutrient availability which were crucial for effective nutrient absorption by the plants.

### 4.2. Responses of Soil Organic Carbon Fractions by Biochar and BM Amendments

As a plant-derived carbon source, POC primarily consists of litter, roots, and undecomposed plant residues, and plays a key role in soil carbon turnover and cycling [18]. In this view, the contents of POC all responded positively to biochar and BM addition, and the beneficial effects were most pronounced under CSB2 + BM treatment. This suggests that biochar combined with BM exhibits a synergistic effect in enhancing soil carbon fractions, demonstrating superior efficacy compared to biochar amendment alone. The POC content under the CSB treatments increased significantly with higher addition rates due to the direct exogenous carbon input from biochar and indirect carbon input from plant growth under biochar addition [37]. Meanwhile, biochar increased POC through contributing actively to soil aggregate formation and soil structure improvement [10,38]. Specifically, we observed a reduction in POC content under the CSB3 + BM treatment, which may be attributed to the facilitated POC transformation to MAOC, as evidenced by the highest MAOC content under CSB3 + BM treatment. This inhibitory effect on soil POC suggested that 4% biochar combined with BM boosted soil carbon converse to a more stable form that is resistant to microbial breakdown because of mineral protection [39].

MAOC is primarily dominated by the mineral adsorption of low-molecular-weight compounds, with a higher proportion of microbial residues that are generally difficult to be decomposed by microorganisms [40]. We found that the application of biochar and BM boosted the content of MAOC and the ratio of MAOC/SOC, and the combination of biochar with BM was advantageous for enhancing MAOC sequestration. Such improvement might be ascribed to the mineral safeguard and biochemical defense, which accounted for the enhanced stability of MAOC. First, biochar and BM could promote organic molecule adsorption onto metal oxides and clay minerals, formulating organo-mineral complexes as a form of physical protection [40,41]. Additionally, biochar increased the carbon content in micro-aggregates and improved carbon storage in micro-aggregates in the long term [42]. The particle size of MAOC is less than 53 μm. Increased soil porosity and improved microaggregates formation were conductive to MAOC accumulation [10]. Second, the microbial activities stimulated by biochar addition promoted the accumulation of microbial biomass and residues through assimilation, which in turn continuously contributed microbial necromass carbon to the soil [41,43]. We found significant positive correlations between MAOC and both microbial and enzyme activities, confirming biochemical protection’s crucial role in MAOC sequestration. Microbial metabolites accelerate the soil aggregation process, and they interact with soil minerals to form MAOC, thereby promoting the stabilization of SOC [43].

As key indicators for evaluating SOC dynamics, DOC, MBC, and RC regulated the supply of nutrients and the turnover of organic carbon. We discovered that DOC showed a marked downward trend as the biochar application rate increased, reducing by 21.94–76.01%. This is consistent with previous studies that reported that DOC content decreased from 193.27 mg·kg^−1^ to 152.95 mg·kg^−1^ in biochar amended soil [44]. Owing to its considerable content of condensed aromatic (recalcitrant) carbon that is highly resistant to microbial decomposition, straw biochar contributed to a marked decline in DOC levels [45]. Additionally, the decreased availability of DOC in soil resulted from functional group protonation and the high adsorption of biochar, which enhanced intermolecular interactions through both proton bridging effects and van der Waals force intensification [12]. Based on the findings of this study, soil MBC was enhanced by 22.13–109.48% following biochar and BM application, especially in the CSB3 + BM treatment. This indicated that the joint use of biochar and BM exerted a more substantial effect on MBC. Biochar and BM application could accelerate soil enzyme activities and microbial growth and activities, thus offering a greater nutrient supply for MBC enhancement [18,45]. This was evidenced by MBC concentrations exhibiting strong relationships with soil nutrients and enzyme and microbial activities in saline-alkali soil. The RC content showed that CSB3+ BM > CSB2 + BM > CSB2> CSB3 > CSB1 + BM > BM > CSB1 > CK. In the relatively higher biochar and BM addition treatments, RC content increased more than the lower biochar addition treatments. These results matched those of Zheng et al. [46], who discovered that adding biochar led to a rise in RC by 18.41–32.31% at 6 and 12 t ha^−1^. This may be because the combined addition of biochar and BM improved the content of aryl and phenolic carbon functional groups, which is the main fraction of RC.

### 4.3. Effects of Biochar and BM Application on Bacterial Community Diversity and Composition

The application of both biochar and BM significantly enhanced soil bacterial diversity in coastal saline-alkali soil, with both the Chao1 index and Shannon index showing significant rises in comparison to the control treatment. Consistent with our findings, Liu et al. [14] reported that the synergistic application of biochar and BM led to more pronounced enhancements in bacterial community diversity within saline-alkaline soil compared to biochar individual treatments. It showed that the soil bacteria community was more susceptible to shifts in the soil environment and variations in the bacterial ability to utilize nutrient substrates [7]. This improvement can be ascribed to the enhanced soil SOC and nutrient contents, offered by biochar, which are accessible to the microbial communities in soil [47]. This was confirmed by the positive correlation between bacterial diversity and SOC, MBC, POC, MAOC, TC, TN, and TP (Figure 6). Moreover, the rise in soil porosity and beneficial microorganisms due to biochar and BM addition safeguards certain microorganisms from predation, thus boosting bacterial diversity [48]. Notably, biochar coupled with BM addition alleviated salt stress to bacterial growth, which had a positive effect on bacteria diversity and activity [14,49].

Biochar and BM amendments also restructured bacterial community composition in coastal saline-alkali soil, triggering significant phylum-level reorganization and genus-level abundance shifts. *Proteobacteria* emerged as the dominant phylum under biochar and BM treatments, exhibiting the highest relative abundance under the CSB2 + BM treatment. The enhancement in soil nutrients and enzyme activities provided a more favorable environment for *Proteobacteria* growth and reproduction [14]. Due to the presence of various pathogenic and nitrogen-fixing bacteria within the *Proteobacteria*, they were capable of mineralizing organic nitrogen and fixing atmospheric nitrogen, which could provide the available nitrogen for plants [8]. *Actinobacteria* showed the highest relative abundances under the CSB3 + BM treatment, suggesting that the boosted nutrient environment was conducive to bacterial growth and proliferation [7,50]. Additionally, *Actinobacteria* participated in degrading recalcitrant SOM forms, potentially contributing to the slow release of soil fertility via SOM decomposition by adding biochar and BM [14]. The greater rise in the relative amount of *Bacillus* under the CSB3 + BM treatment suggested that it could produce more growth hormones and antibacterial substances beneficial to plant growth [51].

### 4.4. The Underlying Biological Mechanisms of Organic Carbon Sequestration and Stability in Biochar and BM Amended Soil

**Figure 7:**
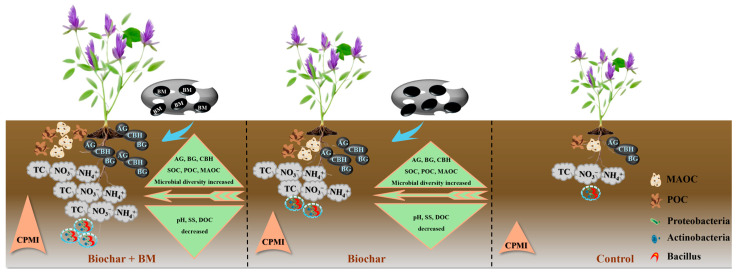
Conceptual framework for the effect of biochar and BM addition on the soil organic carbon composition and stability of coastal saline-alkali soil.

Our results showed that biochar and BM, whether utilized singly or in conjunction with one another, enhanced SOC storage by 14.71–108.02% on coastal saline-alkali soil. This finding converged with the global meta-analysis by Li et al. [45], wherein biochar amendment brought about a 49.4% rise in SOC and the response magnitude governed by biochar properties, biochar application rates, soil properties, and agricultural practices. In this study, the incorporation of biochar and BM led to a more substantial enhancement of SOC content relative to the alone addition of biochar, indicating that the integrated utilization of biochar and BM can trigger a synergistic effect on carbon mineralization and sequestration., as shown in Figure 7. Moreover, the pronounced increased effect of corn straw biochar on SOC also confirmed the previous study which showed the magnitude of the positive effect on SOC observed in crop straw biochar (71.5%) compared to wood biochar addition (41.8%) [12]. Three biological mechanisms were responsible for SOC improvement and sequestration. First, biochar comprised high carbon content and aromatic substances, which can inhibit the microbial breakdown and chemical transformation of SOC, thus storing more SOC and improving its stability [52]. The beneficial microorganisms in BM can accelerate the SOM decomposition and promote the formation of soil aggregates, thereby reducing SOC loss [16]. Second, biochar and BM application enhanced the bacteria diversity and activity, changed the bacteria community composition, increased the functional expression and metabolic activity of dominant microorganisms, further improved plant growth and increased nutrient input to soil, and boosted SOC sequestration [7,12,13]. Third, SOC was positively regulated by bacteria diversity, soil nutrients (TC, NH_4_^+^, and NO_3_^−^), soil carbon enzyme activities (AG, BG, and CBH), and SOC fractions (MAOC, POC, DOC) (Figure 6). A higher rate of biochar addition and BM may improve SOC storage through enhancing carbon enzyme activities and microbial community colonization, which facilitated the conversion of imported exogenous carbon into stable carbon and fostered more MAOC formation [10].

CMPI is widely accepted as a sensitive indicator reflecting the changes in SOC pool and soil quality, and a higher CMI means a more robust SOC pool [53]. Our study revealed that biochar integrated with BM application enhanced CMPI in saline-alkali land, suggesting that biochar and BM improved stable SOC sequestration and improved nutrient cycling functions (Figure 7). Moreover, SOC stability was also impacted by the ratio of MAOC/POC [18]. Notably, biochar integrated with BM application improved the ratio of MAOC/POC, and the ratio of MAOC/POC was positively correlated with SOC (Figure 6). Biochar application has been shown to significantly increase SOC stability by enhancing the stabilization and transformation of the MAOC pool and the rate of MAOC/POC [10]. Notably, CSB3 + BM treatment had a higher rate of MAOC/POC than the other treatments. The 4% biochar combined with BM utilization into saline-alkali soil exerted a more evident optimization of the MAOC contribution to SOC and SOC sequestration. In summary, the synergistic application of biochar and BM could promote SOC stability and sequestration in coastal saline-alkali land by improving the soil nutrient level and SOC fractions, boosting carbon enzyme activities, and increasing soil microbial activity. Further research should conduct long-term field experiments to evaluate the effects of biochar and EM co-application on saline-alkali soil restoration and carbon storage improvement.

## 5. Conclusions

This study explored the effects of applying various amounts of biochar and BM to coastal saline-alkali soil, aiming to quantitatively illustrate the effects of biochar and BM on SOC fractions and sequestration. Our results showed that the application of biochar and BM reduced soil salinity, increased soil nutrient levels and enhanced soil carbon cycle enzyme activities. Additionally, biochar and BM application increased SOC, POC, MAOC, and MBC but decreased DOC in soil. Biochar also increased bacterial diversity and altered community composition, thereby promoting the conversion of external carbon into stable carbon and facilitating the formation of more MAOC. The CBS3+BM treatment enhanced the MAOC/POC ratio and CPMI, which boosted SOC stability and sequestration capacity in coastal saline-alkali land. In summary, 4% biochar addition combined with BM re-duced soil salinity and increased SOC, thereby stimulating microbial growth and enzyme activity, indicating an overall improvement in soil quality.

## Figures and Tables

**Table 1:** Effects of BC and BM and their interaction on (A) soil properties and nutrients, (B) SOC pool fractions, (C) proportions of SOC pool fractions, and (D) soil carbon cycle enzymes.

Variable		BC	BM	BC × BM
(A) Soil properties	pH	2.69	8.44 **	1.19
and nutrients	Soil Salinity	83.31 ***	83.91 ***	22.65 ***
	TC	62.00 ***	108.33 ***	5.41 **
	TN	8.10 ***	21.00 ***	0.29
	AP	79.64 ***	6.49 *	4.89 **
	AK	20.01 ***	37.12 ***	0.49
	NH_4_^+^	23.80 ***	377.51 ***	4.60 *
	NO_3_^−^	29.83 ***	188.93 ***	9.01 ***
(B) SOC pool fractions	SOC	100.15 ***	384.52 ***	8.92 ***
	POC	33.03 ***	419.02 ***	5.54 **
	MBC	39.44 ***	29.65 ***	5.57 **
	MAOC	90.77 ***	202.13 ***	20.05 ***
	DOC	31.36 ***	98.96 ***	0.46
	RC	63.40 ***	76.21 ***	2.69
(C) Proportions of	MAOC/POC	27.48 ***	0.87	20.36 ***
SOC pool fractions	POC/SOC	21.20 ***	0.02	13.58 ***
	MBC/SOC	2.19	19.83 ***	1.00
	MAOC/SOC	21.20 ***	0.02	13.58 ***
	DOC/SOC	62.55 ***	211.07 ***	1.53
	RC/SOC	6.00 **	19.23 ***	0.01
(D) Soil carbon cycle	AG	111.78 ***	317.23 ***	38.93 ***
enzymes	BG	92.26 ***	245.88 ***	24.33 ***
	XG	38.80 ***	78.53 ***	3.46 *
	CBH	61.06 ***	251.88 ***	4.71 *

Note: TC (total carbon, g·kg^−1^); TN (total nitrogen, g·kg^−1^); AP (available phosphate, mg·kg^−1^); AK (available potassium, mg·kg^−1^); SOC (soil organic carbon, g·kg^−1^); MAOC (mineral-associated organic carbon, g·kg^−1^); POC (particulate organic carbon, g·kg^−1^); MBC (microbial biomass carbon, g·kg^−1^); DOC (dissolved organic carbon, g·kg^−1^); RC (inert carbon, g·kg^−1^); AG (α-1,4-glucosidase, nmol·kg^−1^·h^−1^); BG (β-1,4-glucosidase, nmol·kg^−1^·h^−1^); XG (β-Xylosidase, nmol·kg^−1^·h^−1^); CBH (cellulose hydrolase, nmol·kg^−1^·h^−1^); The given are F values of two-way ANOVAs. Asterisks indicate significance levels: *** *p* < 0.001, ** *p* < 0.01, * *p* < 0.05.

**Table 2:** Response of ability of C (CL), lability index (L), carbon pool index (CPI), and carbon pool management index (CMI) to different treatments.

Treatments	CL	L	CPI	CPMI
CK	0.78 ± 0.08 ab	1.00 ± 0.11 ab	1.00 ± 0.03 g	101.03 ± 14.13 d
CSB1	0.72 ± 0.09 ab	0.92 ± 0.12 ab	1.15 ± 0.03 fg	105.98 ± 15.25 cd
CSB2	0.53 ± 0.04 b	0.68 ± 0.05 b	1.25 ± 0.03 ef	85.67 ± 7.32 d
CSB3	0.73 ± 0.05 ab	0.93 ± 0.06 ab	1.41 ± 0.04 cd	132.21 ± 11.07 bcd
BM	1.04 ± 0.11 a	1.32 ± 0.13 a	1.38 ± 0.02 de	182.98 ± 20.24 abc
CSB1 + BM	0.93 ± 0.08 a	1.19 ± 0.11 a	1.55 ± 0.05 bc	185.96 ± 21.73 ab
CSB2 + BM	0.73 ± 0.04 ab	0.92 ± 0.05 ab	1.64 ± 0.02 b	151.76 ± 9.07 bcd
CSB3 + BM	0.97 ± 0.09 a	1.24 ± 0.12 a	2.08 ± 0.03 a	257.23 ± 25.50 a

Note: Different letters indicate significant differences between treatments (*p* < 0.05).

## Data Availability

The original contributions presented in this study are included in the article. Further inquiries can be directed to the corresponding author.
